# Application of UV-Vis Optical Spectroscopy and X-ray Diffraction Methods to Describe the Effect of Alpha-Emitting Radionuclides (Radon) When They Are Detected by Solid-State Film Detectors

**DOI:** 10.3390/polym14132731

**Published:** 2022-07-04

**Authors:** Dana Yerimbetova, Artem Kozlovskiy, Valeriy Stepanenko, Kassym Zhumadilov

**Affiliations:** 1 Engineering Profile Laboratory, L.N. Gumilyov Eurasian National University, Satpayev St., Nur-Sultan 010008, Kazakhstan; dana.erimbetova@mail.ru (D.Y.); kassymzh@yahoo.com (K.Z.); 2 Laboratory of Solid State Physics, The Institute of Nuclear Physics, Ibragimov St., Almaty 050032, Kazakhstan; 3 ASU Innovations, Kh. Dosmukhamedov Atyray University, Studenchesky Ave., Atyrau 060009, Kazakhstan; 4 A.Tsyb Medical Radiological Research Center—Branch of FSBI NMRRC of the Ministry of Health, 249036 Obninsk, Russia; valerifs@yahoo.com

**Keywords:** radon, solid-state nuclear track detector (SSNTD), nitrocellulose detector, optical spectroscopy, X-ray diffraction, alpha-particle detection

## Abstract

This work aims to evaluate the application of optical and X-ray spectroscopy methods to determine the effect of alpha-emitting radionuclides on the properties of solid-state nuclear track detectors (SSNTD) based on nitrocellulose during their detection. The proposed estimation methods are alternative methods to standard technologies, making it possible to determine the concentration of radon and its decay products without the chemical etching of film detectors and subsequent direct counting of the formed latent tracks from interacting particles. During the research, it was found that the use of optical spectroscopy and X-ray diffraction methods makes it possible to qualitatively determine the irradiation effect on changes in the properties of film detectors when α-particles with different energies pass through them. At the same time, a comparison of the data of optical spectroscopy, X-ray diffraction and the visualization of latent tracks after chemical etching made it possible to establish that a part of the registered α-particles in living quarters has an energy of less than 2.5 MeV, which is not enough to pass through the polymer film of the detector, as a result of which well-like tracks are formed. An increase in the intensity of the interference bands in the region above 700 nm and a decrease in the intensity of diffraction reflection characterized the changes in optical transmission. The penetration of the α-particles through the detecting film decreases the film’s transmission capacity, forming an anisotropic change in diffraction reflections associated with a change in the film’s structure and defective fractions distorting the molecular structure.

## 1. Introduction

According to the World Nuclear Association (WNA), Kazakhstan occupies one of the top-ranked places in terms of reserves and production of uranium. At the same time, uranium mining is accompanied by many risks, related to radiation safety and environmental protection [1,2,3,4,5,6]. One of the decay products of uranium is radon-222, which is an invisible radioactive gas that can accumulate and penetrate soil and rocks. Additionally, radon can enter residential premises through microcracks in the foundation, and its easy solubility in water allows it to easily contaminate natural water sources. At the same time, one of the critical tasks for any country is to ensure the radiation safety of the population and compliance with environmental standards, especially near objects with an increased radiation background. The main tasks of monitoring radiation safety are to assess the background values of radiation parameters, including the total activity of alpha-emitting radionuclides in the environment, as well as to analyze the dynamics, identify the causes of changes in the radiation background in the environment, and determine the nature of radiation pollution [7,8,9,10]. As a rule, standard methods for monitoring the radiation background using various detecting devices are used to solve these problems. The most common method for determining radon concentration and its decay products is based on solid-state nuclear track detectors (SSNTD) [11,12,13,14,15].

SSNTD detection is based on determining the passage of heavy charged particles, ionizing the material, thereby causing changes at the molecular level, forming latent tracks along the trajectory of particles in the material, the radius of which is several nanometers [16,17]. At the same time, for their calculation, as a rule, standard methods of the chemical etching of latent tracks in aqueous alkaline solutions are used, which makes it possible to obtain pores with sizes varying from several hundred nanometers to several microns. Such visualization of latent tracks makes it possible to calculate the density of observed tracks by several operations and, with the further use of classical calculation methods, to determine the volumetric activity of radon. However, despite their simplicity, these methods have significant limitations in determining the density of latent tracks at high concentrations of emitters of alpha particles. At high densities of latent tracks, chemical etching, due to the chaotic nature of the hit of registered α-particles in a film detector, can lead to the formation of so-called areas of overlapping latent tracks, which are not visually distinguishable and form one large hole of a complex geometric shape. In this regard, it becomes difficult to determine the irradiation density accurately and, consequently, the volumetric activity of radon, using the calculation formula in which density plays a significant role. Previously, in [18], preliminary results were presented assessing the prospects for using X-ray diffraction analysis and UV-Vis spectroscopy to determine the volumetric activity and density of latent tracks in solid-state nuclear track detectors exposed to an Americium-241 source of alpha particles. In this work, it was shown that the use of these methods—in particular, the determination of changes in deformation contributions, changes in the transmission value, and changes in the value of induced absorption—made it possible to compare the observed changes with the density of visualized tracks on a film detector, and also to determine the lower limit of applicability of these methods for the registration of alpha particles. The central hypothesis underlying the proposed research methods is as follows: When an alpha particle passes through the material of the polymer film, which is a detector, structural and optical changes occur in the material along the trajectory of the alpha particle. The proposed methods and technology for determining volumetric activity will expand the range of applications of solid-state nuclear track detectors, and increase the accuracy of determining the concentration of alpha-emitting radionuclides in environmental objects during long exposure times. It should be noted that the proposed technology has further prospects in the form of the possibility of repeatedly using solid-state nuclear track detectors, since the use of X-ray diffraction analysis and optical spectroscopy does not require the chemical etching of latent tracks.

This article aims to study the prospects for using X-ray diffraction and optical spectroscopy methods to analyze the dose dependence and determine the volumetric activity of radon and its daughter decay products, recorded using solid-state nuclear track detectors based on polymer films during high-dose irradiation or long-term exposure. The relevance of this study is in the development of alternative methods, non-destructive solid-state nuclear track detectors due to chemical etching, the determination of volumetric activity based on changes in structural parameters and optical spectra, and the possibility of determining the accumulated radiation dose in the case of prolonged exposure, in which the direct visual counting of tracks is impossible.

## 2. Experimental Part

Standard solid-state nuclear track detectors, LR-115 type 2, manufactured by Dosirad (France) were chosen for the study. These detectors are 12 µm-thick nitrocellulose polymer films deposited on a 100 µm-thick transparent polyester substrate, with a high detection efficiency of α-particles.

The determination of volumetric activity using these detectors is carried out by the interaction of α-particles with the detector material and the formation of a structurally disordered region, which has significant differences in its properties compared to the main non-irradiated material. Traditionally, these areas under the action of chemical reagents (alkali) are etched in the form of wells or tracks, which can be counted and quantified by visualization.

Two types of premises were chosen as objects of study: residential and basement premises located in two cities (Nur-Sultan and Aktobe). The objects for holding were chosen based on their construction time (both houses were commissioned in the same year), and their location within the city, with access to the basement. Film samples used as detectors of radon decay products were placed in perforated 50 mL plastic containers with free air access. The size of the perforation holes was 5–7 mm. At least 3 films put in containers in one place were used to collect statistics. The measurement error of the visualization of the observed latent tracks after chemical etching did not exceed 3%. This difference is due to the chemical etching of tracks for their direct counting, which is associated with the partial etching of tracks or their small sizes, which cannot be counted directly. In the case of measuring optical spectra and X-ray diffraction patterns, the deviations for different spectra did not exceed 1%. Such a small difference in measurement error, in contrast to direct visualization data, is because all structural changes associated with the passage of alpha particles in the material are recorded for unetched films by optical spectroscopy and X-ray diffraction methods. The choice of basements was due to the possibility of measurements in practically unventilated premises, in which the radon concentration can be significantly higher than in residential premises. Bedrooms with constant daily ventilation, located on the 2nd floor of a residential building, were chosen as residential premises. The exposure time of film detectors was 3432 h, after which the samples were removed and sent for research.

The determination of the optical properties and their changes was carried out by the UV-Vis spectroscopy method, for the implementation of which a Jena Specord-250 BU instrument was used. The spectra were taken in the wavelength range from 550 to 1000 nm, with a step of 1 nm.

X-ray diffraction patterns were obtained on a D8 Advance ECO X-ray diffractometer, Bruker. The diffraction patterns were taken in the range 2θ = 10–40°, with a step of 0.03°, with successive rotation by 10° in the geometry φ = 0–360°. The angular range was determined from the polymer’s diffraction pattern characteristic and the estimated intensity changes in reflections dependent on the impact of α-particles.

The visualization of latent tracks and the acquisition of images of the side cleavages of the films were carried out using a Hitachi TM 3030 scanning electron microscope (SEM).

## 3. Results and Discussion

**Figure 1:**
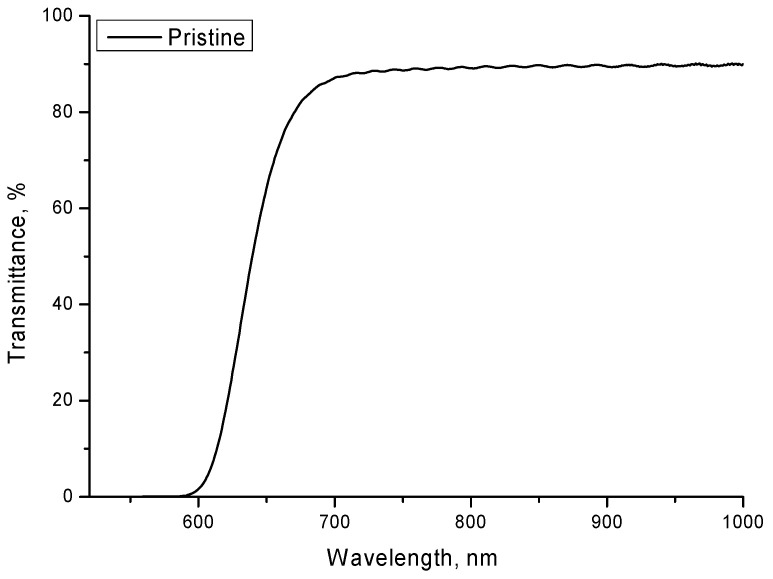
Transmittance change results of initial film LR-115 type 2 (the presented data were obtained with the correction for Fresnel loss, by measuring the reference spectra).

Figure 1 shows the optical spectrum of the films under study in the initial non-irradiated state. The original sample spectrum is characterized by a fundamental absorption edge in the region of 580–600 nm and a transmission value of ~90% in the region above 600 nm. In this case, an interference effect is observed in the region above 700 nm, characteristic of polymer films. The region above 600 nm, characterized by interference bands, was considered for further studies of irradiated films. Under external influences on the film, including irradiation, according to several works, the main changes in the optical properties are characterized by two types of change: a shift in the fundamental absorption edge (redshift), which characterizes the change in the band gap and electron density, and changes in the film transmission in the visible and the IR region, associated with changes in the concentration of defects in the material or the molecular structure, which affects the refractive index and optical density. The shift of the fundamental absorption edge is most pronounced for the irradiation of films with high doses or high-energy ions with high fluences. When irradiated with high-energy ions with high fluences (10^10^ ion/cm^2^ and higher), the formation of areas of overlapping latent tracks is observed and, as follows from several works [19,20], a change in the electron density distribution along the ion motion trajectory in the material.

In the case of the registration of α-particles by these films, the track density is relatively low, while the latent tracks are isolated at a sufficient distance, which excludes the effect of overlap and significant changes in the electron density. As a result, no significant changes in the electron density in the case of isolated latent tracks and the effects associated with them are observed; however, the formed defective regions in the material have a significant effect on the transmission of the films, as well as interference in the region above 600 nm.

**Figure 2:**
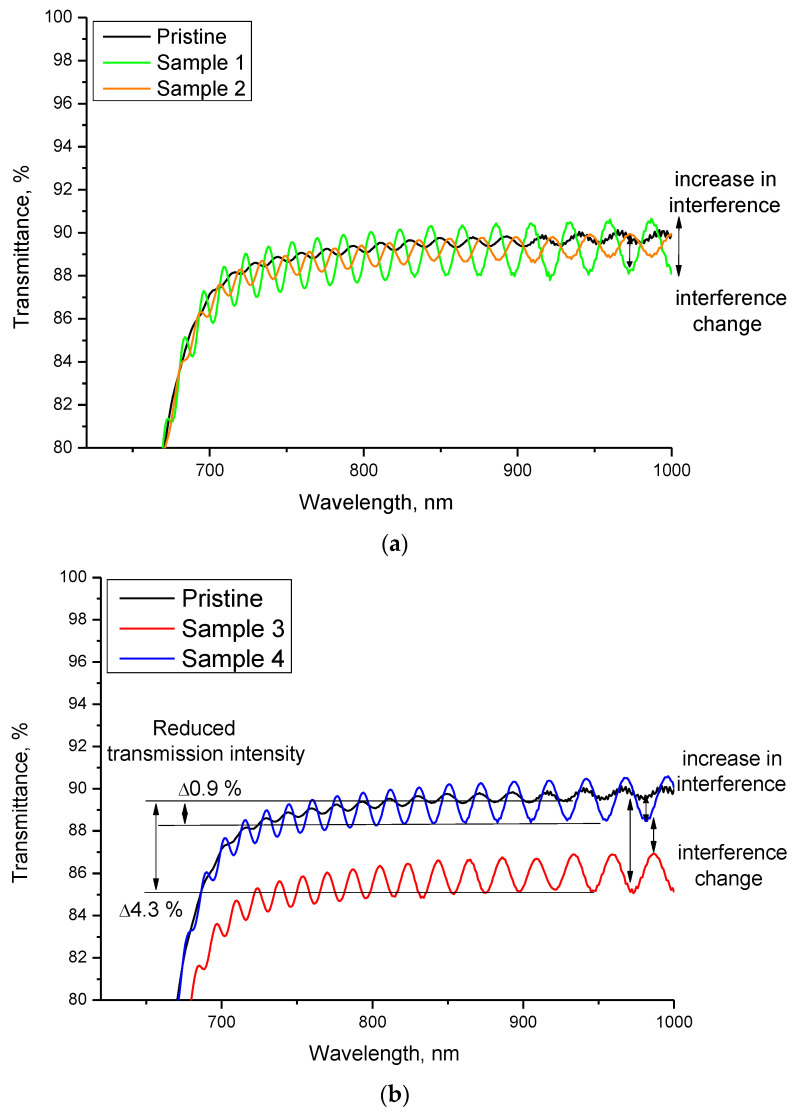
Transmittance results of the studied samples: (**a**) residential premises; (**b**) basements.

Figure 2 shows the UV-Vis spectra of LR-115 type 2 film samples used as detectors in residential areas (Figure 2a) and basements (Figure 2b). The samples were placed according to GOST for measurements and, after exposure, were studied both by optical spectroscopy and X-ray diffraction, and by the classical method of the visual calculation of track density after chemical etching of the films. There are significant differences in the changes in the transmission value for residential and basement premises in terms of UV-Vis spectra. For residential premises, the main changes are associated with changes in the intensity of the interference bands, and their period (interference step). Moreover, compared with the initial spectra, the differences are noticeable for different samples, although a decrease in the transmission value has not been established. This behavior may be due to the small value of the accumulated dose, and the low radon concentration.

In the case of basements, the differences in the optical spectra are more pronounced. Sample №4 exhibited an increase in the intensity of interference and a ~0.9% decrease in transmission in the range of 700–1000 nm. For sample №3, the reduction in transmission was more than 4%, while a change in the interference period was also observed. Such a change in the optical spectra can only be associated with an increase in the accumulated dose and a high density of latent tracks from α-particles. Such significant differences between basements and residential premises are because in basements, in which there is practically no ventilation, the concentration of radon and its daughter decay products can be significantly higher than in residential premises. At the same time, in both cases, optical methods for determining changes in films subjected to irradiation are quite sensitive both to small changes associated with low activity and in the case of significant radiation doses. The nature of the changes in the optical spectra indicates not only a high sensitivity, but also the possibility of qualitatively assessing the effect of alpha particles on films, and a comparison of the results with the values of the track density calculated after chemical etching will make it possible to determine the dose dependences of changes in the optical spectra.

**Figure 3:**
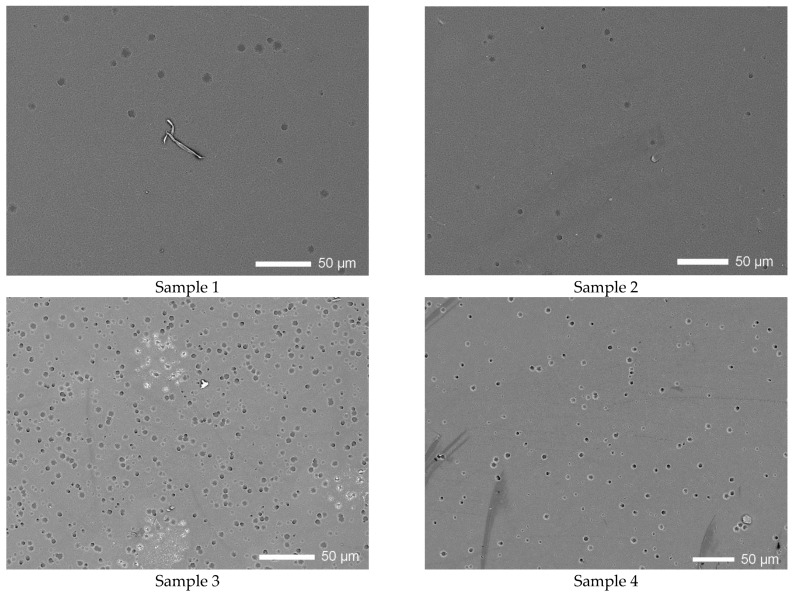
Surface morphology images of SSNTD films after chemical etching to visualize latent tracks.

Figure 3 shows SEM images of the samples under study after chemical etching to visualize the latent tracks of α-particles during their registration by the detector. As can be seen from the presented data, in the case of samples placed in residential premises, there is a small number of registered latent tracks, and some of the tracks are much smaller in size, and of a shape characteristic of unetched well-shaped tracks. At the same time, differences in the density of registered latent tracks were also established, which indicates a different accumulated dose by detectors and different concentrations of radon in the premises.

For the basement samples (Sample 3 and Sample 4), the density of the recorded latent tracks is much higher, which indicates a higher activity of radon and its decay products.

**Figure 4:**
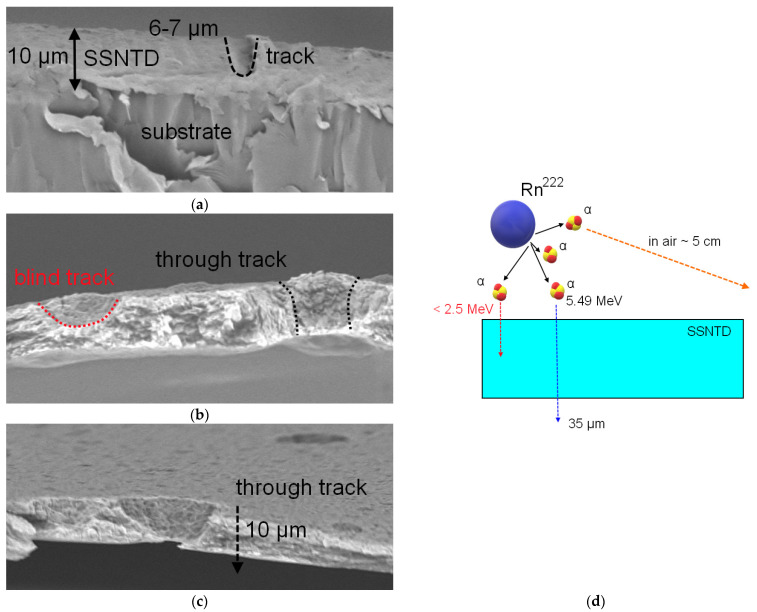
SEM images of side cleavages of the studied film samples: (**a**) sample 1; (**b**) sample 4; (**c**) sample 3; (**d**) schematic representation of the detection of α-particles by film detectors.

Figure 4 shows the results of the SEM images of side cleavages of the investigated SSNTD films after chemical etching, reflecting the geometry of latent tracks from α-particles. When analyzing the images of the film surface, it was found that some of the latent tracks have visual differences, both in diameter and in geometry, which are typical for unetched tracks of shallow depth. In this regard, it was suggested that when film detectors detect α-particles, some of the α-particles have significantly lower energy, which is not enough to completely pass through the thickness of the detecting layer (12 μm). According to estimates, the path length of α-particles with an energy of 5.5 MeV in a polymer film is more than 30 μm, and in the air, it is more than 5 cm. In this case, for the incomplete penetration of films by α-particles, their energy should be less than 2.5 MeV. As can be seen from the results of the side cleavage of samples from living quarters, most of the latent tracks are unetched wells, 6–10 µm deep, which indicates that most of the registered α-particles have an energy of less than 2.5 MeV, which is not enough to penetrate through the films.

In the case of basements for sample №3, through tracks are observed, indicating that the energies of α-particles were sufficient to penetrate the film. In the case of sample №4, both well-like tracks and through tracks were registered. Such a difference in the geometry of recorded latent tracks from α-particles can be due to the difference in the energies of particles emitted by radon, and the radon concentration in the premises. In the case of high radon concentration, the number of emitted α-particles is quite large, leading to the detection of many high-energy α-particles. In the case of residential premises, the concentration of radon is much lower due to constant air circulation and remoteness from the ground (measurements were made in rooms above one floor). Unetched well-like latent tracks can be explained by small changes in the optical spectra for residential premises, and for sample №4, for which the presence of unetched latent tracks was also recorded. In the case of such an interpretation, because small changes in the optical spectra are associated with detecting low-energy α-particles, it can be concluded that changes in the intensity of interference bands are associated with small energy losses of α-particles with an initial energy of less than 2.5 MeV. In this case, when the energy of α-particles is sufficient to pass through the entire thickness of the detector film, changes are observed in the film transmission value, which was observed for samples №3 and №4. With an increase in the detected particle density, a decrease in the transmittance of the films is observed, which is due to radiation damage, and a change in the damaged layer electron density. The reduction in transmittance with the increase in the density of latent tracks has good agreement with the results of polymer films irradiation with high-energy ions. In the case of heavy ion irradiation, when the irradiation density increases, changes in optical properties are observed due to a change in the fundamental absorption edge (change in electron density) and a decrease in transmission (an increase in the absorption and formation of defective inclusions in the irradiated polymer). Because of the low irradiation densities of α-particles (see the data in Table 1), no change in the fundamental absorption edge was found for the samples under study, which is also in good agreement with the results of [21,22], according to which significant changes in the fundamental absorption edge are observed at sufficiently high irradiation fluences (above 10^9^ ion/cm^2^). At the same time, the change in the transmission value, in most cases, is associated with changes in the concentration of defective inclusions or structural changes caused by irradiation.

The most effective way to register changes is the X-ray diffraction method, which allows one to determine the degree of irradiation effect on the material properties from the intensity of diffraction reflections and their changes [23,24].

Table 1 presents the results of determining the values of volumetric radon activity for the samples under study on the various premises. The determination of the volumetric activity of radon was carried out using Formula (1):

(1) $$C=\frac{\rho -\rho_{0}}{KT},$$

where *ρ* is the density of the tracks of alpha particles, track/cm^2^; *ρ*_0_ is the intrinsic background level of the track detector, track/cm^2^; *T* is the duration of the detector exposure, h; and *K* is the calibration factor of the track detector (transition coefficient from the track density value to the volumetric activity value), track·m^3^/Bq·h·cm^2^. In this work, the calibration coefficient of the LR-115 type 2 detector was taken to be *K* = 0.9 track·m^3^/Bq·h·cm^2^, as was experimentally established in a previous study for this type of track detector [25]. For measurements of radon concentration in rooms, it is recommended to take into account the balance factor between radon and its daughter products as F = 0.4 [26].

**Figure 5:**
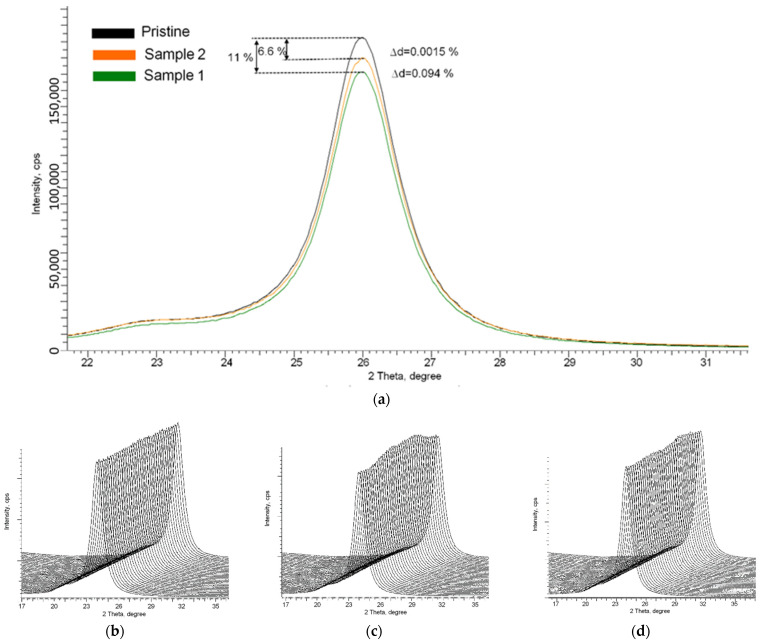
X-ray diffraction patterns of the studied samples of SSNTD films placed in residential premises: (**a**) comparative diffraction patterns of samples after detection of α-particles; representation of X-ray diffraction patterns in survey geometry φ = 0–360°: (**b**) initial sample; (**c**) sample 2; (**d**) sample 1.

Figure 5 shows the results of the X-ray diffraction of samples placed on residential premises (Sample 1 and Sample 2). X-ray diffraction patterns were obtained in the Bragg–Brentano geometry in the angular range of 2θ = 10–40° and characterized by the presence of a diffraction reflection in the region of 2θ = 24–27°, which is characteristic of the polymer and describes its structural properties. As is known from X-ray diffraction analysis, changes in the intensity and shape of diffraction reflections due to external influences characterize structural changes associated with the formation of defects, changes in the molecular structure of a substance, or the deformation of crystalline or chemical bonds. Moreover, by analyzing changes in the shape and intensity of reflections, and changes in the maxima position, one can evaluate the dynamics of structural parameters and qualitatively or quantitatively assess the degree of influence of external effects on the material properties, including irradiation.

According to the presented data of X-ray diffraction patterns, the main changes in the irradiated samples are a decrease in the intensity of reflections relative to the initial intensity value, as well as a shift in the position of the maximum, the value of which corresponds to the interplanar distance between atomic planes (d). In the case of sample №2, the change in intensity was more than 6%, and the shift value *∆*d = 0.0015%, while for sample №1, the change in intensity (*∆*I) and *∆*d was 11% and 0.094%, respectively. The observed changes indicate a change in the structural properties of the films, as well as tensile strains arising in them caused by irradiation with α-particles. In this case, in contrast to optical changes, X-ray diffraction data are more informative and reflect changes in the structure associated with its deformation, and the formation of defective regions along the trajectory of α-particles in the substance. As shown in [23,24,27], X-ray diffraction patterns in the geometry φ = 0–360° make it possible to estimate the contribution of irradiation and the degree of its isotropy, including the isotropy of distortions caused by irradiation. In the case of the original non-irradiated samples, the diffraction pattern obtained in the φ = 0–360° geometry indicates the isotropy of the structure properties. For irradiated samples, as shown in Figure 5c,d, there is a change in intensities with the presence of characteristic maxima and minima, which indicates the effect of anisotropic distortion caused by the interaction of registered α-particles and the molecular structure of the films.

**Figure 6:**
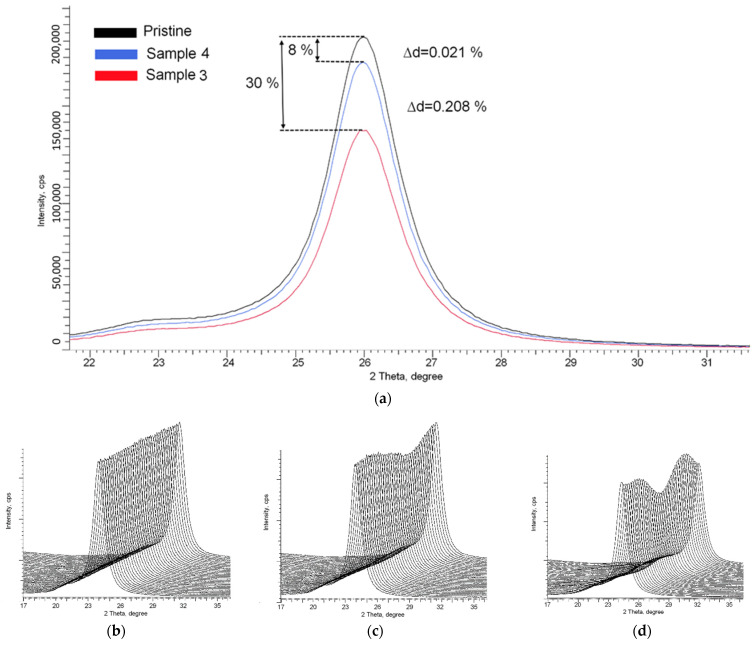
X-ray diffraction patterns of the studied samples of SSNTD films placed in the basement: (**a**) comparative diffraction patterns of samples after detection of α-particles; representation of X-ray diffraction patterns in survey geometry φ = 0–360°: (**b**) initial sample; (**c**) sample 4; (**d**) sample 3.

Figure 6 shows the X-ray diffraction of film samples placed in the basement. According to the obtained data, as in the case of the results of optical spectroscopy, the changes in X-ray diffraction patterns are more pronounced for basements than for residential premises. The maximum decrease in the intensity of X-ray reflections is about 30%, and in the case of survey geometry φ = 0–360°, a strong anisotropy of the intensity of reflections is observed depending on the angular position of the survey. The presence of asymmetry indicates a strong distortion of the molecular structure of the polymer caused by the accumulation of radiation damage from α-particles. Such a strong distortion of diffraction reflections can be because, in the case of basements, the number of registered α-particles that form through latent tracks is much greater than in the case of residential premises. As a result, many latent tracks from α-particles introduce significant distortions into the properties of the detecting film, which are reflected in the anisotropy of diffraction reflections.

Thus, by analyzing X-ray diffraction results, we can draw the following conclusions. When registering α-particles, in the case of low radon concentrations, the X-ray diffraction method makes it possible to establish with high accuracy the effect of α-particles on the molecular structure of polymer films. Such an impact makes it possible to assess the presence of registered α-particles qualitatively, and the use of a survey in the geometry φ = 0–360° by estimating anisotropy allows a qualitative assessment of the energy range of registered α-particles. At the same time, using the method of assessing the degree of radiation damage by determining the values of *∆*I and *∆*d, it is possible to qualitatively and quantitatively evaluate the accumulated dose using conversion factors or correction factors, the establishment of which requires additional research.

According to the data established and the results of [18], it can be seen that the sensitivity limit for determining the presence of structural and optical measurements using the methods of optical spectroscopy and X-ray diffraction has limitations associated with the observed density of latent tracks, the value of which should be greater than 10^2^–10^3^ track/cm^2^. At given values of track densities, optical spectroscopy and X-ray diffraction methods register changes both in the optical properties of the detectors and in the structural properties, which are expressed in the change in the intensities of X-ray reflections and their shift, and in the case of track densities of more than 10^4^ track/cm^2^, they also manifest themselves in the form of the main diffraction reflection asymmetry, when shot in φ = 0–360° geometry. The presence of asymmetry, as well as a decrease in intensity, indicates the formation of structural changes in the film, an increase in the density of which leads to more pronounced recorded changes, expressed in the form of a decrease in the intensity of reflections, as well as their shift. In the case of optical properties, an increase in the density of tracks formed by the detected particles leads to a decrease in the transmittance, which is most noticeable for basements.

**Figure 7:**
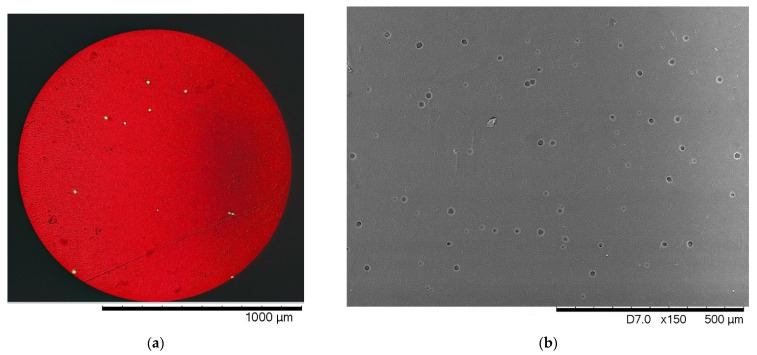
Examples of surface images of the detection film after chemical etching of latent tracks made using various microscopes: (**a**) optical microscope; (**b**) scanning electron microscope (differences in track diameters are clearly visible in the image).

It is worth noting that due to the small number of test samples in this work, it is impossible to determine correction factors for the conversion of dose dependencies and their determination using optical spectroscopy and X-ray diffraction in full, due to the lack of time and dose dependencies obtained on the same objects. The main premise of this work was to demonstrate the possibilities of using X-ray diffraction and optical spectroscopy methods to determine the daughter products of radon decay in residential premises, as an alternative method to the standard method of the direct visual counting of tracks using image processing. At the same time, as can be seen from the analyzed data, the visualization of etched tracks at low densities requires a large amount of statistical data, and a long calculation time due to a small number of observed tracks and their chaotic distribution on the surface. Additionally, according to the presented data for comparing the same film surface (see Figure 7) taken with an optical microscope and a scanning electron microscope, the density of the observed tracks is very dependent on the used optics, which allows detecting tracks. In the case of conventional optical microscopes, detection occurs only for well-etched tracks larger than a few microns in diameter, while when taking images using scanning electron microscopy, the density of the observed tracks is much higher due to the greater microscope resolution, which makes it possible to see incompletely etched tracks, the presence of which was established using side cleavages (see Figure 4). Thus, using standard methods for determining the density of observed tracks, the measurement error and, therefore, the accuracy of determining the dose is very dependent on the instrument used for detection, and the methods used for the chemical etching of tracks. At the same time, optical spectroscopy and X-ray diffraction methods do not require the direct visualization of tracks and allow determining optical and structural changes in irradiated films without preliminary chemical treatment. This makes it possible to use these films for further measurements to determine the timing of the dose set, as well as its change dynamics, depending on the conditions.

## 4. Conclusions

In summary, we can formulate the following conclusions and outline the prospects of this study.

Firstly, during the studies, it was found that UV-Vis spectroscopy and X-ray diffraction methods make it possible to detect α-particles with high accuracy using SSNTD films. At the same time, unlike standard methods that require the chemical etching of films to visualize latent tracks, the use of UV-Vis spectroscopy and X-ray diffraction excludes this procedure.

Secondly, the data obtained from UV-Vis spectroscopy and X-ray diffraction, together with the results of the visualization of the latent tracks of α-particles, showed that in the case of residential premises, the registration of α-particles with an energy of less than 2.5 MeV is observed, which is necessary for the complete passage of the detected α-particle throughout the film thickness. This indicates a low concentration of radon and its daughter decay products in the studied residential premises. In the case of basements, a strong dependence of changes In the optical spectra and X-ray diffraction patterns on the location of the basement was observed, which is expressed in the significant changes in the optical spectra and diffraction patterns associated with deformation contributions caused by the passage of α-particles through the entire thickness of the detecting film, as well as the occurrence of anisotropic distortions, affecting the transmission capacity of the film and its optical density.

Thirdly, the X-ray diffraction results in the survey geometry φ = 0–360° make it possible to estimate the energy contribution of α-particles to structural changes. In the case when α-particles pass through the detecting film, the structural changes caused as a result lead to a considerable anisotropy, and this effect is dependent on the accumulated dose. In the case of the incomplete passage of the detecting layer by α-particles, the value of the anisotropic change in the intensity of diffraction reflections is insignificant.

Further research in this direction will be aimed at the study of the influence of the exposure time of detecting films in various rooms in order to determine the kinetics of dose accumulation, as well as establish coefficients for the transition from optical and X-ray results of changes in film properties to parameters that determine the radon concentration. The proposed methods are alternative methods for determining the accumulated dose, and the use of these methods will allow for the more accurate determination of the volumetric activity of radon at long exposure times, for which standard methods based on direct visualization are not always applicable due to the effect of the overlapping traces of the chemical etching of latent tracks.

The proposed methods for registering the total activity of alpha-emitting radionuclides, as well as determining the accumulated dose value, will increase the accuracy of determining these values for long exposure times and determine the dynamics of changes in the background radiation in selected areas of study.

## Figures and Tables

**Table 1:** Radon volumetric activity data (the measurement data presented in the table were determined by calculating the density of latent tracks and the volumetric activity for all studied films with further determination of the average value and standard deviation).

Sample	Premise Type	Density of Latent Tracks, Tracks/cm^2^	Volumetric Activity, Bq/m^3^
Sample 1	Residential	3409 ± 103	1103.6 ± 16.3
Sample 2		3124 ± 106	1011.4 ± 21.4
Sample 3	Basement	20,300 ± 466	6102.69 ± 32.12
Sample 4		1825 ± 121	590.84 ± 7.18

## Data Availability

Not applicable.
